# The Importance of Exosomal PD-L1 in Cancer Progression and Its Potential as a Therapeutic Target

**DOI:** 10.3390/cells10113247

**Published:** 2021-11-19

**Authors:** Lingxiao Ye, Zhengxin Zhu, Xiaochuan Chen, Haoran Zhang, Jiaqi Huang, Shengxian Gu, Xiaoyin Zhao

**Affiliations:** Lab of Chemical Biology and Molecular Drug Design, Institute of Drug Development & Chemical Biology, College of Pharmaceutical Science, Zhejiang University of Technology, 18 Chaowang Road, Hangzhou 310014, China; lingxiaoye3@gmail.com (L.Y.); zzx101221@163.com (Z.Z.); cxc1999zjut@163.com (X.C.); 201806030229@zjut.edu.cn (H.Z.); 201906030808@zjut.edu.cn (J.H.); gsx479999@163.com (S.G.)

**Keywords:** exosomes, exosomal PD-L1, tumor progression, therapeutic strategy

## Abstract

Binding of programmed cell death ligand 1 (PD-L1) to its receptor programmed cell death protein 1 (PD-1) can lead to the inactivation of cytotoxic T lymphocytes, which is one of the mechanisms for immune escape of tumors. Immunotherapy based on this mechanism has been applied in clinic with some remaining issues such as drug resistance. Exosomal PD-L1 derived from tumor cells is considered to play a key role in mediating drug resistance. Here, the effects of various tumor-derived exosomes and tumor-derived exosomal PD-L1 on tumor progression are summarized and discussed. Researchers have found that high expression of exosomal PD-L1 can inhibit T cell activation in in vitro experiments, but the function of exosomal PD-L1 in vivo remains controversial. In addition, the circulating exosomal PD-L1 has high potential to act as an indicator to evaluate the clinical effect. Moreover, therapeutic strategy targeting exosomal PD-L1 is discussed, such as inhibiting the biogenesis or secretion of exosomes. Besides, some specific methods based on the strategy of inhibiting exosomes are concluded. Further study of exosomal PD-L1 may provide an effective and safe approach for tumor treatment, and targeting exosomal PD-L1 by inhibiting exosomes may be a potential method for tumor treatment.

## 1. Introduction

Programmed cell death protein ligand 1 (PD-L1) is a type I transmembrane protein, which is expressed in many cell types including a variety of tumor cells. Its receptor, programmed cell death protein 1 (PD-1), is an immunoinhibitory receptor preferentially expressed on the surface of immune cells, especially on activated T cells [1]. The binding of PD-L1 to PD-1 inhibits cytotoxic T cell responses, while blockade of this interaction has proven to be an effective approach for various cancers [2]. However, clinical studies have shown that only some patients can achieve complete responses and disease remission. Recent evidence suggests that tumor-derived exosomes may be responsible for immunosuppression of the tumor microenvironment and resistance to anti-PD-L1/PD-1 immune checkpoint therapy [3].

**Figure 1:**
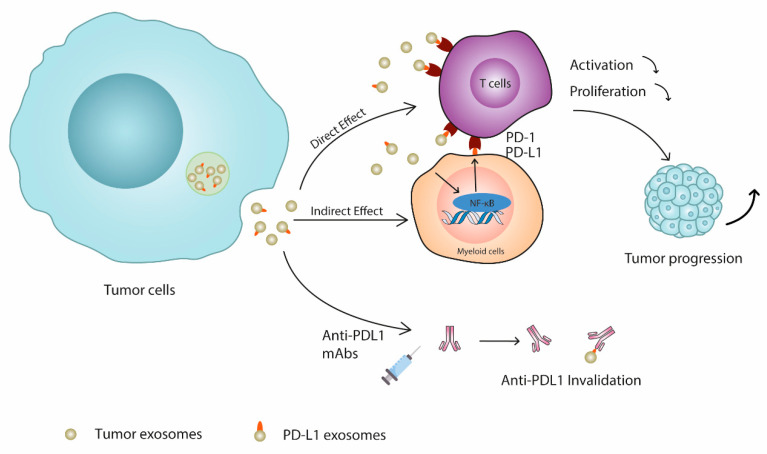
Mechanisms of exosomal PD-L1 mediated immunosuppression. In general, the interaction between exosomal PD-L1 and PD-1 on T cells directly causes immunosuppression. In addition, exosomal PD-L1 up-regulates the expression of PD-L1 in myeloid cells through the NF-κB pathway, makes them transform into myeloid immune cells, and indirectly inhibits the activation and proliferation of T cells. Besides, exosomal PD-L1 can be blocked by anti-PD-L1 mAbs, which may result in a low response rate to anti-PD-L1 mAb treatment. The end point of these mechanisms is T cell inactivation and tumor progression.

Exosomes are extracellular vesicles derived from cells with a 30–150 nm diameter and covered by a lipid bilayer membrane. They exist in almost all body fluids and part of a new type of communication system between cells. The system can carry various cellular molecules that can regulate the physiological morphology of cells and is also closely related to the occurrence of various diseases [4]. Studies have found that tumor derived exosomes can directly inhibit the proliferation of CD8+ T cells, the production of cytokines and cytotoxicity [5]. In addition, studies have shown that the regulatory function of immune cells can be blocked by tumor exosomes, causing the cells to fail to induce T cell activation. Ning et al. reported that tumor exosome-treated dendritic cells (DCs) could inhibit CD4+ T cell proliferation and differentiation. Recent studies have reported that tumor-derived exosomes carrying immune checkpoint proteins can participate in tumor progression, among which exosomal PD-L1 has attracted extensive attention. By blocking exosomal PD-L1, the immune-suppressive effect of tumor exosome-treated DCs could be partially reversed [6]. In addition, tumor cell-derived exosomes can indirectly inhibit anti-tumor immunity by up-regulating the expression of PD-L1 on various immune cells in the tumor microenvironment, thereby creating suitable conditions for the growth of tumor cells, a microenvironment with failed immunity. Immune cells, such as monocytes and macrophages, can inhibit the activation and cytokine production of effector T cells by directly expressing PD-L1 or secreting PD-L1-positive exosomes when stimulated by persistent inflammation or tumor exosomes [7]. Moreover, Chen et al. reported that the level of pre-treatment circulating exosomal PD-L1 was related to the response rate of anti-PD-L1 monoclonal antibodies (mAbs), suggesting the importance of exosomal PD-L1 for anti-PD-1/PD-L1 tumor therapy [5]. In summary, the direct impact of exosomal PD-L1 on T cells or its regulation of T cells through other immune cells may be an important mechanism for tumor cell immune escape (Figure 1). This review summarizes the role of exosomes derived from different types of cancer cells in tumor progression, focuses on the immunosuppressive effect and mechanism of exosomal PD-L1, and proposes strategies for using exosomes as tumor therapeutic targets.

## 2. Effects of Exosomes on Tumor Progression

### 2.1. Effects of Different Tumor-Derived Exosomes on Tumor Progression

In recent years, it has been found that exosomes play an important role in the progression of different tumors (Table 1). Most studies have reported that tumor-derived exosomes promote tumor cell proliferation and migration, but a few studies have reported that tumor-derived exosomes inhibit tumor progression. Exosomes usually play their roles by acting on certain pathways or cells. Moreover, exosomes are important vectors of miRNAs, which can transfer miRNAs to nearby cells or distant target cells, where they participate in the regulation of signaling pathways, thereby influencing tumor progression. Therefore, exosomes are potentially to be developed as a new therapeutic and prognostic target.

### 2.2. Exosomes Delivered from Various Tumors

#### 2.2.1. Gastric Cancer

Increasing evidence indicates that exosomes play important roles in gastric cancer development and progression. Liu and colleagues found that gastric cancer (GC) tumor cell-derived exosomes resulted in immunosuppressive tumor microenvironments in the lungs of mice, with increased CD4+ T cell and myeloid-derived suppressor cell (MDSC) frequencies, and decreased CD8+ T cell and natural killer cells (NK cells) frequencies [8]. Moreover, Zhang et al. demonstrated that tumor exosomes can induce autophagy and neutrophil tumor-promoting activation by the HMGB1/TLR4/NF-κB signaling pathway [9]. Exosomes can carry a variety of biological molecules and regulate local and distal cell communication [27]. For instance, Yang and coworkers reported that exosomes carrying miR-130a could promote angiogenesis and tumor growth [10].

#### 2.2.2. Hepatocellular Carcinoma

Due to the high incidence rate and mortality of hepatocellular carcinoma (HCC), the prognosis of patients is generally not satisfactory. Therefore, there is an urgent need for new treatments to inhibit metastasis and recurrence. Rao et al. indicated that HCC-derived exosomes could effectively carry HCC antigen to stimulate DCs mediated immune response, ultimately improve the tumor microenvironment and induce tumor inhibition [11]. Subsequently, the mechanism studies from Chen et al. showed that exosomes induce epithelial–mesenchymal transition (EMT) through MAPK/ERK signal transduction pathway to alter the biology of hepatoma [12]. More recently, Han et al. reported that HCC-derived exosomes could decrease the cytotoxicity of T-cells and NK cells, promoting the immuno-suppressive M2 macrophages, N2 neutrophils, and Bregs [13].

#### 2.2.3. Leukemia

In recent years, more and more studies have shown that the involvement of exosomes in the treatment of leukemia is a potential therapeutic strategy. In a study by Huang and collaborators, by silencing TGF-β1, leukemia derived exosomes targeted DCs to induce potent anti-leukemic immunity in a mouse model [14]. Subsequent experiments proved that TGF-β1-silenced leukemia cell-derived exosomes could promote the proliferation of CD4+ T cells and the secretion of Th1 cytokines, and more effectively stimulate cytotoxic T lymphocyte (CTL) response and NK cytotoxicity, which can inhibit tumor growth and prolong the survival of mice [15]. Besides, Chen et al. suggested that exosomes from acute myeloid leukemia cells could propel bone marrow stromal cells (BMSCs) to produce IL-8 to increase leukemia cell resistance [16]. Recently, Haque et al. indicated that inhibitor therapy of exosomal miR-181a inhibited leukemia cell proliferation in vitro [17].

#### 2.2.4. Glioma

Exosomes have also been implicated in the progression of glioma. In a study by Bu and collaborators, tumor-derived exosomes loaded DCs have been shown to induce CD8+ CTL response to their tumor cells and kill autologous glioma cells in vitro [18]. Xu and colleagues revealed a study that exosomal miR-375 promoted the proliferation and invasion of glioma cells by activating the CTGF-EGFR oncogenic pathway [19].

#### 2.2.5. Melanoma

Melanoma cell-derived exosomes (MTEX) have an immunosuppressive effect on immune cells. A previous study by Sharma et al. demonstrated that MTEX inhibited the expression of CD69 in CD8+ T cells and down-regulated the expression of NKG2D in NK cells [20]. In addition, Boussadia et al. indicated that melanoma cells might obtain the ability of migration and invasion in an acidic environment due to the metastasis of metastatic exosome protein, which is conducive to cell movement and angiogenesis [21].

#### 2.2.6. Renal Cell Carcinoma

Most patients with renal cell carcinoma (RCC) are resistant to conventional radiotherapy and chemotherapy [28]. Exosome involvement may be an effective treatment strategy for RCC. Xu et al. showed that the Renca cell-derived exosome (RDE) stimulated CD8+ T cells to produce specific cytotoxicity affecting autologous tumor cells through the Fas ligand (FasL)/Fas signaling pathway, and the combination of CD8+ T cells stimulated by RDE with GM-CSF and IL-12 enhanced the anti-tumor effect [22].

#### 2.2.7. Lung Cancer

Exosomal miRNA and lncRNA play a vital role in the pathogenesis of lung cancer [29]. Sun et al. recently demonstrated that exosomal miR-106b can be served as a novel marker to increase the expression of MMP-2 and MMP-9 and target phosphatase and tensin homolog (PTEN) and down-regulate its expression to enhance the migration and invasion ability of lung cancer cells [23].

#### 2.2.8. Breast Cancer

Breast cancer has become the most common cancer worldwide. Wang and collaborators suggested that exosomal miR-1910-3p down-regulated MTMR3, activated NF-κB and Wnt/β-catenin signaling pathways, and ultimately promoted breast cancer progression [24].

#### 2.2.9. Cervical Squamous Cell Carcinoma

Cervical squamous cell carcinoma (CSCC) is one of the most common malignant tumors in women [30]. A previous study by Zhou et al. reported that exosomal miR-221-3p secreted by CSCC is transferred to human lymphatic endothelial cells (HLECs) and then promotes lymphangiogenesis and lymphatic metastasis by downregulating vasohibin-1(VASH1) [25]. Additionally, exosomal miR-221-3p is also involved in promoting angiogenesis [26].

## 3. The Effects of Exosomal PD-L1

### 3.1. Effect of Tumor Derived Exosomal PD-L1 on Tumors

In recent years, the significant role of exosomal PD-L1 in tumor progression has attracted intense attention from researchers. Unlike soluble PD-L1 and cell surface PD-L1, exosomal PD-L1 can interact with PD-1 in a remote manner and inhibit the activity of T cells. Current studies on the function of exosomal PD-L1 derived from melanoma [5], gastric cancer [31], non-small cell lung cancer [32,33], osteosarcoma [34], and other tumors have demonstrated that exosomal PD-L1 could inhibit T cell activity and accelerate tumor growth through interaction with PD-1 in vitro. In an in vitro study of melanoma [5], compared with the control group, MEL624 cell derived exosomal PD-L1 reduced the proliferation of human peripheral blood CD8+ T cells by 40%, and the expressions of Ki-67 and Granzyme B decreased by 30%.

Although its ability of tumor inhibition in vitro has been confirmed, the role of exosomal PD-L1 on tumor in vivo remains controversial. Clinical data regarding melanoma [5], head and neck cancer [35], gastric cancer [36], non-small cell lung cancer [33,37], osteosarcoma [34], and other cancers have shown that the high level of exosomal PD-L1 is positively correlated with disease progressions, such as advanced tumor stage, large tumor size, lymph node-positive, and distal metastasis. However, in two studies on glioblastoma multiforme (GBM), one study by Ricklefs et al. found that mesenchymal subtypes with higher expression of exosomal PD-L1 were more likely to inhibit T cells [38], while the other study by Himes et al. found that GBM derived exosomal PD-L1 did not significantly inhibit T cells [39]. Besides, similar to the above contradiction results, Chen et al. [5] reported that elevated exosomal PD-L1 levels were associated with the metastasis and progression of melanoma, while study by Cordonnier et al. showed that the baseline exosomal PD-L1 levels were not associated with clinical disease progression [40]. In addition, in the same study by Cordonnier et al., PD-1 mAbs pembrolizumab was used to treat melanoma, and it was found that the level of exosomal PD-L1 before administration was negatively correlated with the response rate to pembrolizumab therapy. They also found that increased level of exosomal PD-L1 after treatment indicated a better prognosis. The opposite result reported by Chen showed that the increased level of exosomal PD-L1 after pembrolizumab treatment indicates a worse prognosis. In addition to considering the influence of experimental design on the conflicting results, the different sources of exosomes may also need to be taken into consideration. The above two articles pointed out that there was a certain relationship between the level of exosome PD-L1 and the benefit of treatment, which needs further study to clarify.

### 3.2. Regulation of PD-L1 Expression by Tumor Exosomal PD-L1 in Immune Cells

Tumor exosomes can induce immune cells to express PD-L1, in addition to expressing PD-L1 themselves, which indirectly inhibits T cell activation. Under physiological conditions, immune cells such as regulatory T cells, myeloid-derived suppressor cells, monocytes, and neutrophils can modulate immune response by expressing PD-L1. Under pathological conditions, tumor exosomes can induce the expression of PD-L1 on these immune cells, enhancing the inhibition of local and even systemic immunity. The mechanism by which tumor exosomes induce PD-L1 expression on the immune cells remains unclear, but studies in GBM suggest that the indirectly immunosuppressive effect through up-regulating PD-L1 in the immune cells may be stronger than the directly immunosuppressive effect by tumor exosomal PD-L1 [39]. In addition to GBM, gastric cancer derived exosomes had been reported to up-regulate the expression of PD-L1 in neutrophils via the STAT3 pathway [31], indicating that the mechanism by which tumor exosomes affect the expression of PD-L1 in immune cells may be quite common. Since the microenvironment in tumor tissues is more complex than the culture conditions in vitro, it is not enough to only study the inhibitory function of tumor exosomal PD-L1, but the role of immune cells expressing PD-L1 should also be considered seriously. Literatures related to tumor exosomal PD-L1 are listed (Table 2).

## 4. Therapeutic Strategy of Exosomal PD-L1 as Target

Current immunosuppressive therapy and immune checkpoint blockade are focused on the PD-1/PD-L1 pathway, which was proved to correlate with the invasion and metastasis ability of tumor cells. One of the hot topics of immunotherapy is anti-PD-1/PD-L1 therapy. This therapy exhibits a certain effect, but with defects such as individual response, different effects on various tumors [47,48], and resistance [49]. Experiments have shown that exosomal PD-L1 and PD-L1 on the surface of tumor cells have the same topological structure and biological activity, which suggests that targeting exosomal PD-L1 to inhibit tumor progression potentially may be an effective method.

Multiple experiments [33,35,46] have confirmed that exosomal PD-L1 plays an important part in tumor progression and the treatment of anti-PD-L1 antibodies. Besides, the exosomal PD-L1 is believed to play a vital role in the anti-PD-L1 treatment resistance of tumor cells. A possible mechanism concerning the resistance is that the exosomal PD-L1 is highly expressed and competitively binds to anti-PD-L1 antibodies, causing PD-L1 on the surface of tumor cells to be able to bind to the PD-1 receptor of T cells and inhibit T cell function. However, the specific mechanisms still need to be explored.

### 4.1. The Strategies for Targeting Exosomal PD-L1

Targeting exosomal PD-L1 may be beneficial to solve the anti-PD-L1 antibody treatment resistance, which can act as an effective adjuvant therapy. The main effective strategy for targeting exosomal PD-L1 is to eliminate exosomes, which includes inhibiting the biogenesis of exosomes and hindering exosome secretion.

One strategy is to inhibit the biogenesis of exosomes. Knockout or deletion of some essential proteins involved in the process of exosomal biogenesis can inhibit the secretion of exosomal PD-L1. For example, in RAB27a knockout and nSMase2-deficient cell models [46,50], it was found that the knockout or deletion decreased the number of exosomes. Therefore, it may be an effective method to selectively block the substances that participate in the various links of exosomal biogenesis, reducing the expression of exosomal PD-L1. The approach involves regulating genes and proteins, whose specific mechanism is relatively complicated and remains to be explored. The other strategy is to target the secretion of exosomes. Some experimental results [42,51] support that the combination of inhibitors targeting exosomal secretion and anti-PD-L1 antibody therapy has a higher therapeutic effect.

When studying the inhibition of exosomal PD-L1, the researchers found that there is an abscopal effect [46], that is, local (administration site) tumor growth inhibition may lead to distal (non-administration site) tumor suppression. Besides, the immune response is not only systemic, but the duration of action is also long-lasting. In some experiments [46], when the cells treated with exosomal PD-L1 blocking were injected with cancer cells again for some time, the cancer cells were quickly destroyed. It indicates that after being treated with exosomal PD-L1 blocking, the cells have produced a long-term immune response.

Several exosome inhibitors have been developed. For example, tipifarnib has evident exosome inhibitory effect and may be selective for tumor cells [52]. It is worth noting that exosome inhibitors are usually reported to influence the exosomal process of certain tumor cells, but it is still unknown whether they are effective on other types of tumor cells. In addition, safety should be strictly considered when researching and developing exosome secretion inhibitors to avoid immune-related adverse events.

In general, it is necessary to develop exosome inhibitors that selectively target cancer cells as much as possible to improve the anti-tumor effect while reducing the impact on normal cells.

### 4.2. The Specific Methods of Inhibiting Exosomes

There are two common methods regarding the specific inhibition of the occurrence and secretion of exosomes. One method is to use exosome inhibitors to reduce the level of exosomes through different molecules that affect the development, packaging, and secretion of exosomes. There are various types of exosome inhibitors, including those targeting RAB27A, such as Tipifarnib [52], Nexinhib20 [53], and Ketoconazole [52], which have been proved to inhibit the secretion of exosomes. Exosome inhibitors targeting sphingomyelinase, such as GW4869 [54], Manumycin A [55], and Spiroepoxide [56], also have the ability to block the secretion of exosomes. Other types of exosome inhibitors, such as proton pump inhibitors [57], calcium channel blocking agent Ketotifen [58], and Simvastatin [59], have also been found to inhibit the secretion of exosomes but their efficiency exhibits very differently. All these exosome inhibitors mentioned above have shown the ability to block the secretion of exosomes, but more experiments on different tumor cells should be investigated to support the conclusion and their clinical safety remains unsure. In addition, attention should be paid to monitoring the cytotoxicity of exosome inhibitors to normal cells. In addition to newly developed exosome inhibitors, approved drugs can also be used to explore whether they can work on exosomal PD-L1, which may be a more effective and safe way.

Since exosomes have been identified as a tumor progression influencing factor, another method is to separate exosomes from the blood circulation by in vitro ultrafiltration to inhibit the growth of tumor cells. The advantage of this method compared with the chemical drugs is that it can avoid toxic effects on normal cells and drug interactions. Aethlon designed a blood filtration treatment program called adaptive affinity platform technology, whose mechanism of action is similar to kidney dialysis. In this method, the matrix can be formulated according to the purpose, and different affinity agents can be customized to identify and capture the soluble protein in the plasma specifically and then remove it. The safety and effectiveness of this method have been verified in the early experiment [49,60]. Therefore, this method can be used as a method to remove exosomes. Existing experiments [49] have demonstrated that blood purification technology can capture tumor-derived exosomes based on specific molecules on the surface of the exosome. The use of in vitro ultrafiltration and anti-PD-L1 antibody as the matrix can possibly eliminate PD-L1+ exosomes.

However, exosomes also exist in normal cells. Tumor cells can transfer exosomal PD-L1 from PD-L1+ cells to PD-L1− cells [42]. The exosomes of normal cells may be transformed into PD-L1+ cells by tumor cells and separated by ultrafiltration, which causes adverse severe immune reactions. More in-depth preclinical toxicity tests are urgently needed.

## 5. Conclusions and Future Perspectives

**Figure 2:**
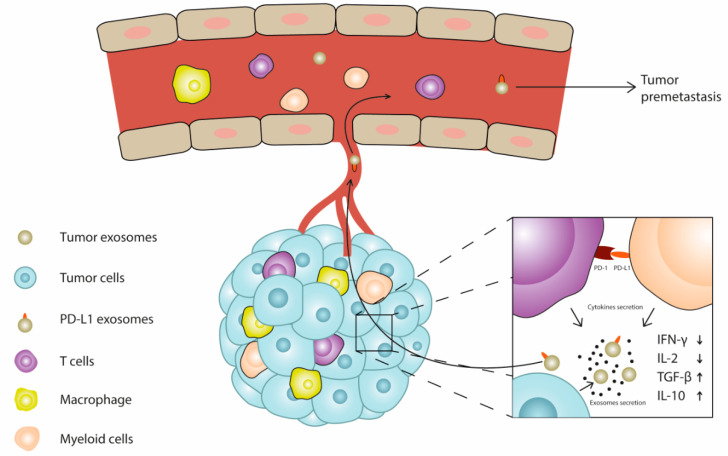
Schematic diagram of the effect of exosomal PD-L1 on tumor microenvironment. At the primary tumor site, T cells interact with PD-L1 positive cells, resulting in immunosuppression, and the types of cytokines released also changed, expanding the impact of immunosuppression. Exosomes carrying PD-L1 cargo are released into the blood and have a similar immunosuppressive effect on premetastatic niche, so that the metastatic tumor can escape the monitoring of the immune system.

This review summarizes current knowledge regarding the role of exosomes and the exosomal PD-L1 in tumor progression, and concludes some therapeutic strategy based on the exosomes. The role of exosomal PD-L1 in in vitro experiments is relatively clear, but there are some contradictions in in vivo experiments. In addition to considering the differences between exosomes from different sources, exosomes can affect immunity in a variety of ways. They can affect immunity not only through the binding of PD-L1 and PD-1, but also through the “cargo”, such as proteins, miRNAs and siRNAs delivered by exosomes. The influence of exosomes on the immune system includes altering the accumulation of immune cells in primary and metastatic site of tumor. In addition, by regulating the release of immunosuppressive factors of immunocytes, exosomal PD-L1 also affects immunity in a wide range, which is not conducive to initiating immunity against tumors. Therefore, focusing on the systemic effects of exosomes on the immune system will also help us understand how exosomes contribute to distant metastasis of tumors (Figure 2). It should be noticed that when discussing the clinical results related to exosomes, the source of plasma circulating exosomes is not limited to tumors. The heterogeneity of the source of exosomes makes it more difficult for us to explore real problems in the human body. Researchers have found that the level of exosomal PD-L1 can be used as a possible clinical biomarker to evaluate clinical results, which also reflects the vital role of exosomal PD-L1 [37,38]. Because of the importance of exosomal PD-L1, we believe that strategies targeting exosomal PD-L1 are effective and promising for tumor treatment, such as inhibiting the occurrence and secretion of exosomes. However, in the actual treatment, the toxicological effects or immune-related adverse reactions that may occur during the treatment, as well as the precise targeting of tumor exosomes remain to be studied and resolved.

Although the role of exosomal PD-L1 in tumor progression has been well studied, the role of other immune checkpoints such as CTLA-4, LAG-3, TIM-3, TIGIT, and CD155 in exosome-based tumor immune regulations have been poorly reported. Gao et al. [61] found that high expression of exosomal TIM-3 and exosomal Galectin-9 in non-small cell lung cancer was positively correlated with several malignant parameters, such as advanced stages, larger size, and more distant tumor metastasis. Qiu et al. [62] reported that exosomal PD-1 in triple-negative breast cancer can interact with PD-L1 on tumor cell surface or exosomal PD-L1, induce PD-L1 internalization and thereby block the interaction between PD-1 and PD-L1, helping recover tumor surveillance via attenuating PD-L1 induced suppression of cytotoxic T cells. The effect of other immune checkpoints mentioned above on exosome-based tumor immune regulation is still unclear and needs to be further explored. We believe that more in-depth research on the role of exosomal PD-L1 and other immune checkpoints in exosome-based tumor immune regulation can lead to effective and safe ways to treat tumors.

## Figures and Tables

**Table 1:** The role of exosomes derived from different tumors.

Tumor Type	Origin of Exosome	Pathway/Targets	miRNA	Recipient Cells	Tumor Progression (In Vitro)	Tumor Progression (In Vivo)	Refs
Gastric cancer	MKN-28, MKN-45 and SGC-7901			CD4+ T cells, MDSC cells, CD8+ T cells, and NK cells	Induced apoptosis of CD8+ T cells and promoted immunosuppression	Promoted lung metastasis of cancer cells in mice	[8]
	BGC8-23, MGC80-3 and SGC-7901	HMGB1/TLR4/NF-κB		Neutrophils	Induced neutrophil autophagy and promoted tumor growth		[9]
	SGC7901 and GES-1	C-MYB	miR-130a		Promoted proliferation and migration of HUVECs	Promoted tumor angiogenesis in mice	[10]
Hepatocellular carcinoma	Hepa1-6			DCs	Induced antigen-specific cytolysis	Improved tumor immune microenvironment in mice	[11]
	MHCC97H, MHCC97L, LM3 et al.	MAPK/ERK			Increased migration and invasion of low-metastatic HCC cells	Promoted recurrence of intrahepatic tumor	[12]
	MHCC97L, HepG2 et al.			T-cells, NK cells, M2 macrophages, N2 neutrophils, and Bregs	Promoted immune-escape and tumor progression		[13]
Leukemia	L1210	TGF-β1		DCs	Inhibited TGF-β1 expression and improved cellular immune function	Inhibited tumor growth and improved survival rate of mice	[14]
	L1210 and p388	Th1		CD4+ T cells, CTL, NK cells, and DCs	Inhibited tumor cells growth	Prolonged survival of mice	[15]
	KG1A, NB4, and MV411			BMSCs	Increased IL-8 expression to increase drug resistance	Increased drug resistance in patients	[16]
	SUP-B15, JM1		miR-181a		Promoted proliferation of leukemia cells		[17]
*Glioma*	Human glioma cells			CTLs	Activated T cells to become CTLs and kill glioma cells.		[18]
	U87, U251, A172 et al.	CTGF-EGFR	miR-375		Activated oncogenic pathway to promote the proliferation of glioma cells	Inhibited proliferation and invasion of glioma by inhibiting exosome secretion	[19]
Melanoma	Human melanoma cells			CD8+ T cells and NK cells	Inhibited CD8+ T cells and induced immunosuppression		[20]
	WM115 and WM266–4				Promoted tumor cells movement and angiogenesis		[21]
Renal Cell Carcinoma	RenCa cells	FasL/Fas		CD8+ T cells	Improved anti-cancer effect by combination with GM-CSF and IL 12	Increased percentage of CD8 + /CD4+ T cells and inhibited growth of tumors	[22]
Lung cancer	SPC-A-1, H358, A549 et al.	PTEN	miR-106b		Increased tumor cells migration and invasion by downregulating PTEN		[23]
Breast cancer	MCF-7 and MDA-MB-231	NF-κB/MTMR3	miR-1910-3p		Promoted tumor cells autophagy and inhibited it apoptosis	Inhibited tumor weight and volume growth	[24]
Cervical squamous cell carcinoma	Siha, Caski, C33a et al.	VASH1	miR-221-3p	HLECs	Induced lymphangiogenesis by inhibiting VASH1 expression	Promoted lymphangiogenesis and lymphatic metastasis	[25]
	SiHa and C33a	THBS2	miR-221-3p	HUVECs	Inhibited angiogenesis by THBS2 overexpression	Promoted tumor growth in mouse models	[26]

**Table 2:** Effects of exosomal PD-L1 from various tumors on T cells and tumors.

Tumor Type	Origin of Exosome	Exosome Characteristic	Recipient	Direct/Indirect Effect	Outcome (In Vitro/In Vivo)	Ref
Melanoma	Human MEL624 cell line	PD-L1+	Human peripheral CD8+ T cells	①	Inhibited proliferation and granzyme-B production; Inhibited IL-2, IFN-γ, and TNF-α secretion	[5]
	Human WM9 cell line	PD-L1+	Human peripheral CD8+ T cells	①	Inhibited activation	[5]
	Murine B16-F10 cell line	PD-L1+	Mouse splenic CD8+ T cells; B16-F1 Tumor-Bearing C57BL/6 mice model	①	Inhibited proliferation and cytotoxicity; Facilitated melanoma growth in vivo	[5]
	RETtransgenic mice skin melanoma cell	PD-L1+	C57BL/6 mice immature myeloid cells	②	Increased immature myeloid cells PD-L1 expression; Suppressed T Cell proliferation	[41]
	Human HT-144 and SK-MEL-28 cell line	PD-L1+	Human CD14+ monocytes	②	Increased CD14+ monocytes PD-L1 expression; Suppressed T Cell proliferation	[41]
	Patients peripheral blood (exosomes isolated by CSPG4 mAbs)	PD-L1+	Human CD8+ T cells and NK cells	①	Decreased CD69 expression; downregulated NKG2D	[20]
	Patients peripheral blood	PD-L1+	patients	③	Level of exosomal PD-L1 positively correlated with disease progression	[40]
Breast carcinoma	Murine 4T1 cell line	PD-L1+	PD-L1 knockout 4T1 Tumor-Bearing BALB/c mice (-) model	③	Facilitated tumor growth in vivo	[42]
Glioblastoma	Human GSC20, GSC267, GSC17 cell line	-	CD14+ monocytes	②	Increased CD14+ monocytes PD-L1 expression and phosphorylation of STAT3	[43]
	Human GSCs cell line (G34, G35, G44, and G157)	PD-L1+	Human CD8+ T cells	①	Inhibited activation; Increased IDO and IL-10 secretion	[38]
	Patients GBM cell	PD-L1+	Peripheral blood T cells and monocytes	②	Did not affect T cells proliferation; Induced PD-L1 in nonclassical monocytes	[39]
Gastric cancer	Patients peripheral blood	PD-L1+	Patients	③	Higher exosomal PD-L1 was associated with poorer prognosis	[36]
	MKN74 cell line	PD-L1+	Peripheral CD8+ T cells	①	Decreased CD69 expression and increased expression PD-1; Increased IL-10 and TGF-β secretion	[36]
	Human BGC-823 cell line	-	Neutrophils	②	Increased Neutrophils PD-L1 expression; Suppressed T Cell Immunity	[31]
	Human MGC-803 and SGC-7901 gastric cancer cell line	-	THP-1 cells	②	Increased THP-1 cells IL-6, TNF-α, CCL2 secretion in NF-κB-dependent manner	[44]
Non-small cell lung carcinoma	Human H1264 cell line	PD-L1+	Human peripheral CD8+ T cells	①	Inhibited proliferation and granzyme-B production	[5]
	Patient’s tissue and peripheral blood	PD-L1+	Patients peripheral CD8+ T cells	①	Inhibited IL-2 and IFN-γ production	[33]
	Patients peripheral blood	PD-L1+	Patients	③	Higher exosomal PD-L1 levels correlated with adverse clinicopathological parameters	[37]
Osteosarcoma	Patients Osteosarcoma cell	PD-L1+; N-cadherin	Patients	③	Higher level of exosomal PD-L1 in metastasis Osteosarcoma patients	[34]
	Osteosarcoma cell line and patients’ serum	PD-L1+; N-cadherin	Human U2OS and 143B cell lines	③	Induced tumor migration and invasion	[34]
Pancreatic ductal adenocarcinoma	Patients’ pancreatic ductal adenocarcinoma cell	PD-L1+	Patients	③	Higher exosomal PD-L1 was associated with PD-L1+ PDAC patients	[45]
Prostate cancer	PC3 cells line	PD-L1+	Raji B cells	①	Inhibited IL-2 production	[46]
	TRAMP-C2 cell line	PD-L1+	Tumor bearing C57BL6/J syngeneic mice	③	Suppressed T cell activation	[46]

PD-L1+: High expression of PD-L1 in tumor exosomes (measured by enzyme linked immunosorbent assay (ELISA), Western blot, flow cytometry, Immunofluorescence labeling, Immunogold labeling) or significantly higher proportion of PD-L1 positive exosomes in tumor patients’ exosomes samples than that in normal samples (measured by flow cytometry). Direct/Indirect Effect: ①: Tumor exosomal PD-L1 directly regulates CD8+ T cells; ②: Tumor exosomal PD-L1 affect T cells indirectly through altering PD-L1 expression and function of other immune cells; ③: Tumor exosomal PD-L1 promoted tumor progression in vivo.

## Data Availability

Not applicable.

## Abbreviations

PD-L1	programmed cell death protein ligand 1
PD-1	programmed cell death protein 1
DCs	dendritic cells
mAbs	monoclonal antibodies
GC	gastric cancer
MDSC	myeloid-derived suppressor cells
NK cells	natural killer cells
HCC	hepatocellular carcinoma
EMT	epithelial-mesenchymal transition
CTL	cytotoxic T lymphocyte
BMSCs	bone marrow stromal cells
MTEX	melanoma cell-derived exosomes
RCC	renal cell carcinoma
RDE	Renca cell-derived exosome
FasL	Fas ligand
PTEN	phosphatase and tensin homolog
CSCC	cervical squamous cell carcinoma
HLECs	human lymphatic endothelial cells
VASH1	vasohibin-1
GBM	Glioblastoma multiforme

## References

[1] Arasanz H., Gato-Cañas M., Zuaz M., Ibañez-Vea M., Breckpot K., Kochan G., Escors D. (2017). PD1 signal transduction pathways in T cells. Oncotarget.

[2] Wang J., Zeng H., Zhang H., Han Y. (2021). The role of exosomal PD-L1 in tumor immunotherapy. Transl. Oncol..

[3] Daassi D., Mahoney K.M., Freeman G.J. (2020). The importance of exosomal PDL1 in tumour immune evasion. Nat. Rev. Immunol..

[4] Maisonneuve C., Tsang D.K.L., Foerster E.G., Robert L.M., Mukherjee T., Prescott D., Tattoli I., Lemire P., Winer D.A., Winer S. (2021). Nod1 promotes colorectal carcinogenesis by regulating the immunosuppressive functions of tumor-infiltrating myeloid cells. Cell Rep..

[5] Chen G., Huang A.C., Zhang W., Zhang G., Wu M., Xu W., Yu Z., Yang J., Wang B., Sun H. (2018). Exosomal PD-L1 contributes to immunosuppression and is associated with anti-PD-1 response. Nature.

[6] Ning Y., Shen K., Wu Q., Sun X., Bai Y., Xie Y., Pan J., Qi C. (2018). Tumor exosomes block dendritic cells maturation to decrease the T cell immune response. Immunol. Lett..

[7] Zhang J., Dang F., Ren J., Wei W. (2018). Biochemical Aspects of PD-L1 Regulation in Cancer Immunotherapy. Trends Biochem. Sci..

[8] Liu J., Wu S., Zheng X., Zheng P., Fu Y., Wu C., Lu B., Ju J., Jiang J. (2020). Immune suppressed tumor microenvironment by exosomes derived from gastric cancer cells via modulating immune functions. Sci. Rep..

[9] Zhang X., Shi H., Yuan X., Jiang P., Qian H., Xu W. (2018). Tumor-derived exosomes induce N2 polarization of neutrophils to promote gastric cancer cell migration. Mol. Cancer.

[10] Yang H., Zhang H., Ge S., Ning T., Bai M., Li J., Li S., Sun W., Deng T., Zhang L. (2018). Exosome-Derived miR-130a Activates Angiogenesis in Gastric Cancer by Targeting C-MYB in Vascular Endothelial Cells. Mol. Ther..

[11] Rao Q., Zuo B., Lu Z., Gao X., You A., Wu C., Du Z., Yin H. (2016). Tumor-derived exosomes elicit tumor suppression in murine hepatocellular carcinoma models and humans in vitro. Hepatology.

[12] Chen L., Guo P., He Y., Chen Z., Chen L., Luo Y., Qi L., Liu Y., Wu Q., Cui Y. (2018). HCC-derived exosomes elicit HCC progression and recurrence by epithelial-mesenchymal transition through MAPK/ERK signalling pathway. Cell Death Dis..

[13] Han Q., Zhao H., Jiang Y., Yin C., Zhang J. (2019). HCC-Derived Exosomes: Critical Player and Target for Cancer Immune Escape. Cells.

[14] Huang F., Wan J., Hao S., Deng X., Chen L., Ma L. (2017). TGF-beta1-silenced leukemia cell-derived exosomes target dendritic cells to induce potent anti-leukemic immunity in a mouse model. Cancer Immunol. Immunother..

[15] Huang F., Wan J., Hu W., Hao S. (2017). Enhancement of Anti-Leukemia Immunity by Leukemia-Derived Exosomes Via Downregulation of TGF-beta1 Expression. Cell Physiol. Biochem..

[16] Chen T., Zhang G., Kong L., Xu S., Wang Y., Dong M. (2019). Leukemia-derived exosomes induced IL-8 production in bone marrow stromal cells to protect the leukemia cells against chemotherapy. Life Sci..

[17] Haque S., Vaiselbuh S.R. (2020). Silencing of Exosomal miR-181a Reverses Pediatric Acute Lymphocytic Leukemia Cell Proliferation. Pharmaceuticals.

[18] Bu N., Wu H., Sun B., Zhang G., Zhan S., Zhang R., Zhou L. (2011). Exosome-loaded dendritic cells elicit tumor-specific CD8+ cytotoxic T cells in patients with glioma. J. Neurooncol..

[19] Xu X., Liu Y., Li Y., Chen H., Zhang Y., Liu J., Deng S., Zheng Y., Sun X., Wang J. (2021). Selective exosome exclusion of miR-375 by glioma cells promotes glioma progression by activating the CTGF-EGFR pathway. J. Exp. Clin. Cancer Res..

[20] Sharma P., Diergaarde B., Ferrone S., Kirkwood J.M., Whiteside T.L. (2020). Melanoma cell-derived exosomes in plasma of melanoma patients suppress functions of immune effector cells. Sci. Rep..

[21] Boussadia Z., Lamberti J., Mattei F., Pizzi E., Puglisi R., Zanetti C., Pasquini L., Fratini F., Fantozzi L., Felicetti F. (2018). Acidic microenvironment plays a key role in human melanoma progression through a sustained exosome mediated transfer of clinically relevant metastatic molecules. J. Exp. Clin. Cancer Res..

[22] Xu H.Y., Li N., Yao N., Xu X.F., Wang H.X., Liu X.Y., Zhang Y. (2019). CD8+ T cells stimulated by exosomes derived from RenCa cells mediate specific immune responses through the FasL/Fas signaling pathway and, combined with GMCSF and IL12, enhance the antirenal cortical adenocarcinoma effect. Oncol. Rep..

[23] Sun S., Chen H., Xu C., Zhang Y., Zhang Q., Chen L., Ding Q., Deng Z. (2020). Exosomal miR-106b serves as a novel marker for lung cancer and promotes cancer metastasis via targeting PTEN. Life Sci..

[24] Wang B., Mao J.H., Wang B.Y., Wang L.X., Wen H.Y., Xu L.J., Fu J.X., Yang H. (2020). Exosomal miR-1910-3p promotes proliferation, metastasis, and autophagy of breast cancer cells by targeting MTMR3 and activating the NF-kappaB signaling pathway. Cancer Lett..

[25] Zhou C.F., Ma J., Huang L., Yi H.Y., Zhang Y.M., Wu X.G., Yan R.M., Liang L., Zhong M., Yu Y.H. (2019). Cervical squamous cell carcinoma-secreted exosomal miR-221-3p promotes lymphangiogenesis and lymphatic metastasis by targeting VASH1. Oncogene.

[26] Wu X.G., Zhou C.F., Zhang Y.M., Yan R.M., Wei W.F., Chen X.J., Yi H.Y., Liang L.J., Fan L.S., Liang L. (2019). Cancer-derived exosomal miR-221-3p promotes angiogenesis by targeting THBS2 in cervical squamous cell carcinoma. Angiogenesis.

[27] Fu M., Gu J., Jiang P., Qian H., Xu W., Zhang X. (2019). Exosomes in gastric cancer: Roles, mechanisms, and applications. Mol. Cancer.

[28] Ljungberg B., Bensalah K., Canfield S., Dabestani S., Hofmann F., Hora M., Kuczyk M.A., Lam T., Marconi L., Merseburger A.S. (2015). EAU guidelines on renal cell carcinoma: 2014 update. Eur. Urol..

[29] Li Y., Yin Z., Fan J., Zhang S., Yang W. (2019). The roles of exosomal miRNAs and lncRNAs in lung diseases. Signal Transduct. Target. Ther..

[30] Koh W.J., Abu-Rustum N.R., Bean S., Bradley K., Campos S.M., Cho K.R., Chon H.S., Chu C., Clark R., Cohn D. (2019). Cervical Cancer, Version 3.2019, NCCN Clinical Practice Guidelines in Oncology. J. Natl. Compr. Cancer Netw..

[31] Shi Y., Zhang J., Mao Z., Jiang H., Liu W., Shi H., Ji R., Xu W., Qian H., Zhang X. (2020). Extracellular Vesicles From Gastric Cancer Cells Induce PD-L1 Expression on Neutrophils to Suppress T-Cell Immunity. Front. Oncol..

[32] Hong W., Xue M., Jiang J., Zhang Y., Gao X. (2020). Circular RNA circ-CPA4/ let-7 miRNA/PD-L1 axis regulates cell growth, stemness, drug resistance and immune evasion in non-small cell lung cancer (NSCLC). J. Exp. Clin. Cancer Res..

[33] Kim D.H., Kim H., Choi Y.J., Kim S.Y., Lee J.E., Sung K.J., Sung Y.H., Pack C.G., Jung M.K., Han B. (2019). Exosomal PD-L1 promotes tumor growth through immune escape in non-small cell lung cancer. Exp. Mol. Med..

[34] Wang J., Zhang H., Sun X., Wang X., Ren T., Huang Y., Zhang R., Zheng B., Guo W. (2020). Exosomal PD-L1 and N-cadherin predict pulmonary metastasis progression for osteosarcoma patients. J. Nanobiotechnol..

[35] Theodoraki M.N., Yerneni S.S., Hoffmann T.K., Gooding W.E., Whiteside T.L. (2018). Clinical Significance of PD-L1(+) Exosomes in Plasma of Head and Neck Cancer Patients. Clin. Cancer Res..

[36] Fan Y., Che X., Qu J., Hou K., Wen T., Li Z., Li C., Wang S., Xu L., Liu Y. (2019). Exosomal PD-L1 Retains Immunosuppressive Activity and is Associated with Gastric Cancer Prognosis. Ann. Surg. Oncol..

[37] Li C., Li C., Zhi C., Liang W., Wang X., Chen X., Lv T., Shen Q., Song Y., Lin D. (2019). Clinical significance of PD-L1 expression in serum-derived exosomes in NSCLC patients. J. Transl. Med..

[38] Ricklefs F.L., Alayo Q., Krenzlin H., Mahmoud A.B., Speranza M.C., Nakashima H., Hayes J.L., Lee K., Balaj L., Passaro C. (2018). Immune evasion mediated by PD-L1 on glioblastoma-derived extracellular vesicles. Sci. Adv..

[39] Himes B.T., Peterson T.E., de Mooij T., Garcia L.M.C., Jung M.Y., Uhm S., Yan D., Tyson J., Jin-Lee H.J., Parney D. (2020). The role of extracellular vesicles and PD-L1 in glioblastoma-mediated immunosuppressive monocyte induction. Neuro. Oncol..

[40] Cordonnier M., Nardin C., Chanteloup G., Derangere V., Algros M.P., Arnould L., Garrido C., Aubin F., Gobbo J. (2020). Tracking the evolution of circulating exosomal-PD-L1 to monitor melanoma patients. J. Extracell. Vesicles.

[41] Fleming V., Hu X., Weller C., Weber R., Groth C., Riester Z., Huser L., Sun Q., Nagibin V., Kirschning C. (2019). Melanoma Extracellular Vesicles Generate Immunosuppressive Myeloid Cells by Upregulating PD-L1 via TLR4 Signaling. Cancer Res..

[42] Yang Y., Li C.W., Chan L.C., Wei Y., Hsu J.M., Xia W., Cha J.H., Hou J., Hsu J.L., Sun L. (2018). Exosomal PD-L1 harbors active defense function to suppress T cell killing of breast cancer cells and promote tumor growth. Cell Res..

[43] Gabrusiewicz K., Li X., Wei J., Hashimoto Y., Marisetty A.L., Ott M., Wang F., Hawke D., Yu J., Healy L.M. (2018). Glioblastoma stem cell-derived exosomes induce M2 macrophages and PD-L1 expression on human monocytes. Oncoimmunology.

[44] Wu L., Zhang X., Zhang B., Shi H., Yuan X., Sun Y., Pan Z., Qian H., Xu W. (2016). Exosomes derived from gastric cancer cells activate NF-kappaB pathway in macrophages to promote cancer progression. Tumour Biol..

[45] Lux A., Kahlert C., Grutzmann R., Pilarsky C. (2019). c-Met and PD-L1 on Circulating Exosomes as Diagnostic and Prognostic Markers for Pancreatic Cancer. Int. J. Mol. Sci..

[46] Poggio M., Hu T.Y., Pai C.C., Chu B., Belair C.D., Chang A., Montabana E., Lang U.E., Fu Q., Fong L. (2019). Suppression of Exosomal PD-L1 Induces Systemic Anti-tumor Immunity and Memory. Cell.

[47] Page D.B., Postow M.A., Callahan M.K., Allison J.P., Wolchok J.D. (2014). Immune Modulation in Cancer with Antibodies. Ann. Rev. Med..

[48] Goswami S., Aparicio A., Subudhi S.K. (2016). Immune Checkpoint Therapies in Prostate Cancer. Cancer J..

[49] Marleau A.M., Chen C.S., Joyce J.A., Tullis R.H. (2012). Exosome removal as a therapeutic adjuvant in cancer. J. Transl. Med..

[50] Ostrowski M., Carmo N.B., Krumeich S., Fanget I., Raposo G., Savina A., Moita C.F., Schauer K., Hume A.N., Freitas R.P. (2010). Rab27a and Rab27b control different steps of the exosome secretion pathway. Nat. Cell Biol..

[51] Liu S.-Y., Huang W.-C., Yeh H.-I., Ko C.-C., Shieh H.-R., Hung C.-L., Chen T.-Y., Chen Y.-J. (2019). Sequential Blockade of PD-1 and PD-L1 Causes Fulminant Cardiotoxicity-From Case Report to Mouse Model Validation. Cancers.

[52] Datta A., Kim H., McGee L., Johnson A.E., Talwar S., Marugan J., Southall N., Hu X., Lal M., Mondal D. (2018). High-throughput screening identified selective inhibitors of exosome biogenesis and secretion: A drug repurposing strategy for advanced cancer. Sci. Rep..

[53] Johnson J.L., Ramadass M., He J., Brown S.J., Zhang J.Z., Abgaryan L., Biris N., Gavathiotis E., Rosen H., Catz S.D. (2016). Identification of Neutrophil Exocytosis Inhibitors (Nexinhibs), Small Molecule Inhibitors of Neutrophil Exocytosis and Inflammation DRUGGABILITY OF THE SMALL GTPase Rab27a. J. Biol. Chem..

[54] Lallemand T., Rouahi M., Swiader A., Grazide M.H., Geoffre N., Alayrac P., Recazens E., Coste A., Salvayre R., Negre-Salvayre A. (2018). nSMase2 (Type 2-Neutral Sphingomyelinase) Deficiency or Inhibition by GW4869 Reduces Inflammation and Atherosclerosis in Apoe(-/-) Mice. Arterioscler. Thromb. Vasc. Biol..

[55] Datta A., Kim H., Lal M., McGee L., Johnson A., Moustafa A.A., Jones J.C., Mondal D., Ferrer M., Abdel-Mageed A.B. (2017). Manumycin A suppresses exosome biogenesis and secretion via targeted inhibition of Ras/Raf/ERK1/2 signaling and hnRNP H1 in castration-resistant prostate cancer cells. Cancer Lett..

[56] Takahashi A., Okada R., Nagao K., Kawamata Y., Hanyu A., Yoshimoto S., Takasugi M., Watanabe S., Kanemaki M.T., Obuse C. (2017). Exosomes maintain cellular homeostasis by excreting harmful DNA from cells. Nat. Commun..

[57] Federici C., Petrucci F., Caimi S., Cesolini A., Logozzi M., Borghi M., D’Ilio S., Lugini L., Violante N., Azzarito T. (2014). Exosome Release and Low pH Belong to a Framework of Resistance of Human Melanoma Cells to Cisplatin. PLoS ONE.

[58] Khan F.M., Saleh E., Alawadhi H., Harati R., Zimmermann W.H., El-Awady R. (2018). Inhibition of exosome release by ketotifen enhances sensitivity of cancer cells to doxorubicin. Cancer Biol. Ther..

[59] Kulshreshtha A., Singh S., Ahmad M., Khanna K., Ahmad T., Agrawal A., Ghosh B. (2019). Simvastatin mediates inhibition of exosome synthesis, localization and secretion via multicomponent interventions. Sci. Rep..

[60] Tullis R.H., Duffin R.P., Handley H.H., Sodhi P., Menon J., Joyce J.A., Kher V. (2009). Reduction of Hepatitis C Virus Using Lectin Affinity Plasmapheresis in Dialysis Patients. Blood Purificat..

[61] Gao J.W., Qiu X.Y., Li X.Y., Fan H., Zhang F., Lv T.F., Song Y. (2018). Expression profiles and clinical value of plasma exosomal Tim-3 and Galectin-9 in non-small cell lung cancer. Biochem. Biophys. Res. Commun..

[62] Qiu Y.F., Yang Y., Yang R.Y., Liu C.X., Hsu J.M., Jiang Z., Sun L.L., Wei Y.K., Li C.W., Yu D.H. (2021). Activated T cell-derived exosomal PD-1 attenuates PD-L1-induced immune dysfunction in triple-negative breast cancer. Oncogene.

